# Customizable Hydrogel Coating of ECM-Based Microtissues for Improved Cell Retention and Tissue Integrity

**DOI:** 10.3390/gels10080515

**Published:** 2024-08-05

**Authors:** Shani Elgin, Eric Silberman, Assaf Shapira, Tal Dvir

**Affiliations:** 1 The Shmunis School of Biomedicine and Cancer Research, Faculty of Life Sciences, Tel Aviv University, Tel Aviv 6997801, Israel; 2 The Sagol Center for Regenerative Biotechnology, Tel Aviv University, Tel Aviv 6997801, Israel; 3 Tel Aviv University Center for Nanoscience & Nanotechnology, Tel Aviv University, Tel Aviv 6997801, Israel; 4 Department of Biomedical Engineering, Faculty of Engineering, Tel Aviv University, Tel Aviv 6997801, Israel; 5 Sagol School for Neuroscience, Tel Aviv University, Tel Aviv 6997801, Israel

**Keywords:** microfluidics, ECM-based hydrogel, nanoparticles, microtissues, hydrogels

## Abstract

Overcoming the oxygen diffusion limit of approximately 200 µm remains one of the most significant and intractable challenges to be overcome in tissue engineering. The fabrication of hydrogel microtissues and their assembly into larger structures may provide a solution, though these constructs are not without their own drawbacks; namely, these hydrogels are rapidly degraded in vivo, and cells delivered via microtissues are quickly expelled from the area of action. Here, we report the development of an easily customized protocol for creating a protective, biocompatible hydrogel barrier around microtissues. We show that calcium carbonate nanoparticles embedded within an ECM-based microtissue diffuse outwards and, when then exposed to a solution of alginate, can be used to generate a coated layer around the tissue. We further show that this technique can be fine-tuned by adjusting numerous parameters, granting us full control over the thickness of the hydrogel coating layer. The microtissues’ protective hydrogel functioned as hypothesized in both in vitro and in vivo testing by preventing the cells inside the tissue from escaping and protecting the microdroplets against external degradation. This technology may provide microtissues with customized properties for use as sources of regenerative therapies.

## 1. Introduction

Perhaps the most significant challenge that must be addressed in the field of tissue engineering is overcoming the oxygen diffusion limit of approximately 200 µm. In native tissues, a dense and complex vascular network fully penetrates the entire structure [1], such that cells are rarely found more than 100 µm away from the nearest capillary [2]. During gestation, the vasculature grows jointly within the developing tissues to ensure that the oxygen needs of all cells continue to be met, even as the organism grows. However, without the benefits of natural embryonic development, tissue engineering must find alternative approaches to overcome the diffusion limitation of oxygen and develop tissues thicker than a few hundred microns.

Many strategies have been proposed to deliver oxygen to cells within thick tissues. Strategies include using endothelial cells and allowing them to naturally form blood vessels [3,4], producing tissues via advanced fabrication techniques (such as lithography and 3D printing) that contain hollow lumens through which cell culture medium can be perfused [5], and designing oxygen-producing scaffolds that generate oxygen from within [6,7]. Another strategy that has gained attention is encapsulating cells within micrometric hydrogel particles, or “microgels”.

Microgels provide several distinct benefits over traditional hydrogels [8,9,10]. First, microgels synergistically combine multiple mechanisms that promote oxygen diffusion. One method by which microgels mitigate hypoxia is by conglomerating to form macroscopic structures with large interstitial volumes that fill the void between particles. Because blood, cell growth medium, or serum can easily fill this entire volume, the diffusion distance required of oxygen is limited to the radius of a single particle [8,11]. At the same time, the large surface-area-to-volume ratio of spheres maximizes the diffusion of oxygen into the individual particles [11]. Besides the benefits that relate to mass transport, microgels provide several other advantages. For example, individual particles from within a macrostructure can be separated with minimal force. As a result, hydrogels composed of microdroplets almost always demonstrate shear thinning behavior, which makes them suitable for delivery via minimally invasive procedures [10]. Additionally, microgels encapsulating different cell types can be assembled in a modular fashion to easily create complex tissues [12]. This strategy can be further enhanced by combining microgels with a suitable technology (such as 3D printing) to provide total control over the placement of cells within the tissue [13,14,15].

Despite their obvious benefits, microgels also suffer from a distinct drawback. Namely, the same factors that promote nutrient diffusion also lead to rapid degradation [16]. In fact, microgels suffer from both the outward movement of encapsulated cells away from the implantation site, termed “cell escape” [17], and the inward flux of macrophages and their associated foreign body response. Multiple techniques have been employed to overcome these challenges, including centering cells within microparticles to retard their escape [18], manipulating the size of the microparticles [19], and developing core–shell microparticles, often based on synthetic polymers that cells cannot easily penetrate [20].

Our lab has previously developed a microfluidic system for encapsulating cells within a micrometric hydrogel derived from native extracellular matrix (ECM) [9]. There, we developed a protocol for the microfluidic system that led to precisely controlled cell encapsulation and high cell viability and functionality. The hydrogel was selected for its biological relevance [21] and the microfluidic process for its high repeatability and throughput.

Here, we sought to enhance the microgels’ stability by generating a protective shell around the microparticles. To that end, we encapsulated cells and nanoparticles of calcium carbonate (^np^CaCO_3_) within the ECM-based hydrogel and used the aforementioned microfluidic system to generate microtissues containing ^np^CaCO_3_. When these microtissues were immersed in a solution of sodium alginate, the ^np^CaCO_3_ diffused out of the ECM-based hydrogel and interacted with the alginate molecules in the immediate vicinity of the tissue to form an alginate-based hydrogel capsule around it. Importantly, the formation of this alginate shell was highly tunable, and it could be controlled by altering several different parameters individually or in parallel. We demonstrated the ability to control the thickness of the alginate hydrogel layer by controlling multiple parameters, including the type of alginate used, its concentration, the concentration of the ^np^CaCO_3_, and the time allowed for the reaction. Microtissues protected in this way were then assessed both in vitro and in vivo. While the alginate coating did not in any way impair cell viability, it did retard cell escape and protect the microgel from cell-based and enzymatic cleavage. This technology was ultimately demonstrated in vivo, and we showed that the presence of the alginate coating led to enhanced cell retention and tissue integrity at the injection site.

## 2. Results and Discussion

In order to formulate a protective coating for microtissues formed from an ECM-based hydrogel, we decided to work with alginate. Alginate is well-documented as a biomaterial owing to its low cost and overall biocompatibility [22]. Additionally, the crosslinking of alginate is orthogonal to that of the ECM-based hydrogel, which allows for each hydrogel to be crosslinked independently of the others [23].

An alginate coating around the ECM-based microgels was generated using an internal gelation strategy [24]. In this method, non-soluble calcium is preloaded in the form of calcium carbonate (CaCO_3_), which is insoluble at physiological pH. However, because CaCO_3_ readily dissolves in acidic environments, lowering the pH leads to the dissolution of the salt and the subsequent crosslinking of the alginate. While this general strategy is well known, several considerations were taken into account when adapting this procedure for use as a coating technique for microtissues.

First, in order to localize the crosslinking to the surface of the microtissues, CaCO_3_ was loaded into the microgels, rather than the alginate solution. This forced us to find a solution that would minimize the volume occupied by the CaCO_3_, so as not to crowd out the cells. Additionally, it was important that the presence of the insoluble crystals not change the rheological properties of the hydrogel and thus disrupt the microfluidic process. Second, because this technique must be relevant for cell-laden microtissues, we sought to minimize the microgels’ exposure to the acidic alginate environment. For these reasons, we chose to work with nanoparticles of CaCO_3_ (^np^CaCO_3_), which, owing to their extremely high surface-area-to-volume ratio, dissolve rapidly upon exposure to a low pH, minimizing the time required for the reaction to occur [25,26].

**Figure 1:**
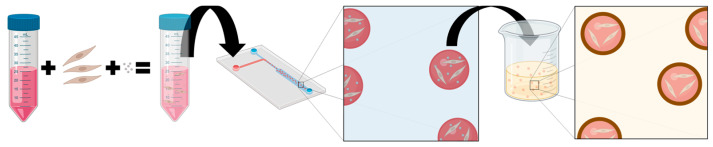
Overall schematic. Cells (brown) and nanoparticles of CaCO_3_ (gray) were encapsulated within an ECM-based hydrogel (red), and a microfluidic system was used to generate microtissues. When the microdroplets were subsequently suspended in a solution of sodium alginate (yellow), the calcium ions crosslinked the alginate and generated a customizable protective shell around the microgel. Created with BioRender.com.

**Figure 2:**
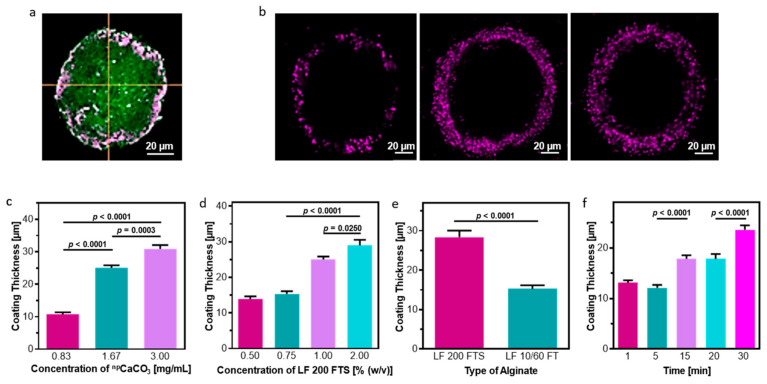
Controlling the Alginate Capsule. (**a**) The 3D confocal imaging revealed that the alginate shell completely surrounded the microdroplet from all sides. For visualization, FITC liposomes were incorporated in the ECM-based hydrogel and alginate was tagged with fluorescent moieties. (**b**) Representative images of alginate barriers with varying thickness and integrity. For visualization, fluorescent microparticles were incorporated in the alginate layer. (**c**) The thickness of the alginate capsule varies proportionally with the concentration of ^np^CaCO_3_ used. (**d**) Increasing the concentration of alginate in which the microdroplets were submersed increases the thickness of the capsule. (**e**) Using longer alginate chains (Protanal^®^ LF 200 FTS vs. Protanal^®^ LF 10/60 FT) led to an increase in capsule thickness. (**f**) Increasing the time during which the reaction was allowed to proceed led to an increase in the thickness of the alginate layer. Scale bars = 20 µm.

We therefore devised a two-step process for the creation of ECM-based microtissues with an alginate coating (Figure 1). First, cells were encapsulated within an ECM-based hydrogel, and then ^np^CaCO_3_ were suspended within the gel. This tissue precursor was then run through a custom-made microfluidic system to generate microgels containing ^np^CaCO_3_. The microfluidic system generates a water-in-oil emulsion in which the hydrogel is the aqueous component. By separately modulating the flow rates of the hydrogel and the surrounding oil phase, it is possible to control the size of the collected droplets [8]. After being collected, the microgels were incubated at 37 °C for 15 min to allow them to thermally gel, at which point the surfactants that had been used to form the droplets were removed by washing. Second, the microgels were immersed in a slightly acidified (pH = 6) solution of sodium alginate. This step was performed on ice in order to both minimize the negative impacts of the acidic pH on the cells and to provide better control over the crosslinking kinetics of the alginate. This protocol led to a robust crosslinking, and an alginate shell was visible around the microdroplets even after repeated washings (Figure 2a).

In order to maximize the versatility of this system, we next undertook a thorough study of the factors controlling the formation of the alginate coating (Figure 2b). Although any one factor might be enough to control the alginate hydrogel layer, by assessing the impact of multiple factors, we can more thoroughly customize the protocol and expand its range of utility. Unsurprisingly, the concentration of ^np^CaCO_3_ incorporated in the hydrogel had a significant impact on the thickness of the alginate coating (Figure 2c). Even at concentrations as low as 0.83 mg ^np^CaCO_3_ per mL hydrogel, an alginate coating with a thickness of 10.73 ± 0.62 µm could be observed.

One of the benefits of using alginate as a biomaterial is that alginate is not, in fact, a single molecule, but rather a class of polymers that vary in length and composition depending on the species of algae from which the material is collected [22]. It is often possible, therefore, to fine-tune a process based on alginate by simply varying the species of algae used. In order to assess the impact of alginate type on our coating protocol, we used both Protanal^®^ LF 200 FTS and Protanal^®^ LF 10/60 FT. Protanal^®^ LF 200 FTS is composed of longer polymeric chains than other forms of alginate and forms significantly more viscous solutions. The reason for the increased viscosity is that the extended chains form significant interactions between themselves, and we hypothesized that these intermolecular forces would likewise act to increase the thickness of the coating when applied to a microtissue. As predicted, the use of the longer Protanal^®^ LF 200 FTS chains led to the formation of a thicker coating (28.39 ± 6.80 µm as compared to 15.25 ± 4.29 µm) around the microgels (Figure 2d).

Alongside the dependence on the concentration of ^np^CaCO_3_, the coating thickness was investigated as a function of the concentration of alginate (Figure 2e). While varying the concentration of alginate below 0.75% did not affect the coating, using concentrations of 1% or 2% led to the creation of a significantly thicker shell around the microgel.

Finally, we investigated our ability to control the thickness of the alginate coating by exposing the microdroplets to acidic alginate for variable lengths of time (Figure 2f). Within one minute of exposure, a discernible coating of 13.10 ± 2.85 µm was already present around the microgel. After 30 min of exposure, the coating thickness had increased to 23.59 ± 5.61 µm. In order to preserve cell viability, the coating procedures were not conducted for time periods exceeding 30 min.

**Figure 3:**
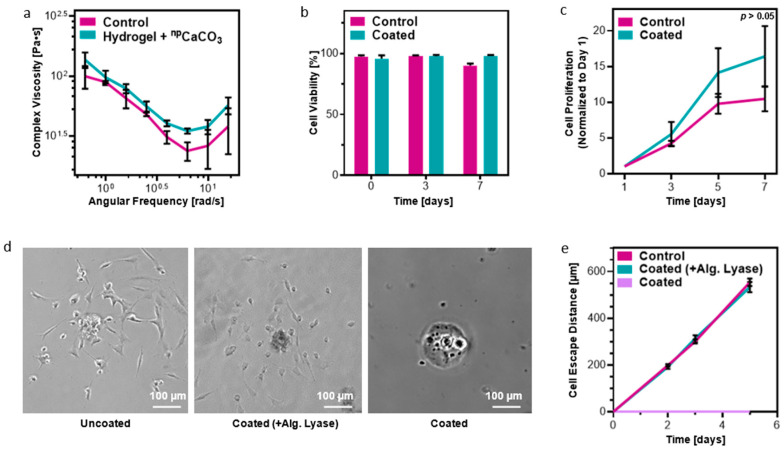
Functionality and biocompatibility of the alginate coating. (**a**) The addition of ^np^CaCO_3_ caused no significant changes in the rheological properties of the ECM-based hydrogel. (**b**) The cell-coating protocol had no negative impact on cell viability, and, indeed, showed a positive increase in cell viability after a week of cultivation. (**c**) The presence of the alginate coating did not impair cell viability. In fact, because the coating acted to protect the microtissue, coated microtissues remained larger and thus were able to accommodate larger and more active cell populations. (**d**) Representative images showing cells that had escaped from the droplet and adhered to the well plate on Day 3 in both uncoated (left) and coated-then-enzymatically-degraded (center) droplets. Droplets with an intact alginate barrier showed negligible cell escape (right). Scale bars = 100 µm. (**e**) The presence of the coating almost entirely halted the ability of the cells to migrate out of and away from the microtissue. The impact of the alginate was further verified by enzymatically degrading the alginate, which led to unimpaired “cell escape”.

Having developed a robust protocol for customizing the thickness of the alginate layer, we next sought to determine the implications of the coating on the behavior of cells encapsulated within the microtissues. First, as it is well known that the mechanical properties of a cell’s microenvironment can significantly impact the cell’s functionality [27,28,29,30,31,32], the rheological characteristics of the ^np^CaCO_3_-infused ECM-based hydrogel were investigated. In our work, the rheological properties of this hydrogel are doubly important as they can both impact the microfluidic process as well as the ability of the encapsulated cells to mature properly. As shown, the addition of the ^np^CaCO_3_ did not lead to any significant changes in the rheological behavior of the hydrogel (Figure 3a). Next, cells were encapsulated within the hydrogels and the entire protocol—the addition of ^np^CaCO_3_, the microfluidic generation of microtissues, and the formation of the alginate coating—was performed. As a proof-of-concept, we chose to work with fibroblasts as a model system to study cell viability, proliferation, and migration. It was observed that the coating protocol did not cause cell death immediately following the treatment, nor in the three days following (Figure 3b). One week after treatment, cell viability decreased slightly for non-coated microtissues, while microtissues that had received the alginate treatment had slightly higher cell viability. This trend was also observed in a PrestoBlue™ assay for cell metabolism (Figure 3c). We attribute the impaired cell viability and proliferation in uncoated droplets to the degradation of the droplets. Whereas the microtissues with an alginate barrier maintained their full volume over the course of the experiment, the non-coated microtissues shrank significantly. Therefore, the remaining cells become significantly denser over time, likely causing a contact-inhibition effect.

In addition, the alginate barrier’s ability to prevent “cell escape” was assessed. While cells encapsulated in non-coated microdroplets migrated 110.3 ± 2.58 µm per day, cells in protected microdroplets showed minimal migration over the course of five days. As an additional test, to ensure that the difference was due to the alginate coating and not to another factor that may have occurred during the crosslinking process, coated droplets were treated with alginate lyase. Alginate lyase is known to cleave alginate chains without harming cells [33]. After treatment with alginate lyase, cells encapsulated in droplets that had a protected layer were suddenly able to “escape” the microtissue. Furthermore, their migration proceeded at precisely the same rate (106.9 ± 3.53 µm per day) as cells that had never undergone the alginate-coating protocol (Figure 3d,e).

**Figure 4:**
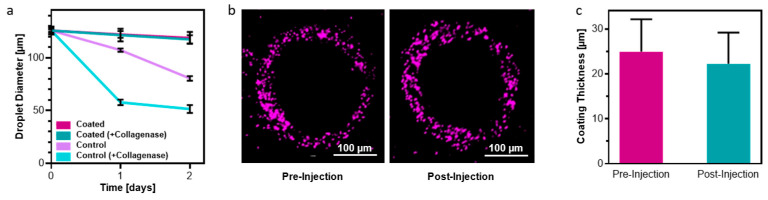
Integrity of alginate barrier. (**a**) The alginate barrier prevented water molecules and even collagenase enzymes from degrading the hydrogel, while unprotected droplets rapidly degraded. (**b**) Representative images showing the thickness and integrity of the alginate shell before (left) and after (right) being injected through a hypodermic needle. Scale bar = 100 µm. (**c**) Quantification of the barrier integrity showed no significant change as a result of injection.

Having ascertained that the presence of an alginate coating not only did not harm cells, but in fact led to greater retention and viability, the behavior of the coating itself was investigated. First, microdroplets were exposed to collagenase to mimic the degradation processes that would occur in vivo, and the degradation kinetics of the ECM-based hydrogel was measured (Figure 4a). Droplets without a protective alginate layer rapidly decreased in size, with a reduction in the average droplet diameter to only 45.9 ± 1.06% of its original diameter over the course of 24 h. Interestingly, even without collagenase, unprotected droplets gradually decreased in size over the course of 48 h. This suggests that water molecules gradually pry apart the biopolymers that compose the ECM-based hydrogel even in the absence of relevant degradation enzymes. On the other hand, droplets protected by a layer of alginate showed no decrease in diameter over the course of the experiment. This demonstrates that the alginate shell acted as an effective two-way barrier, both preventing the ECM-based hydrogel’s biopolymers from washing away, even if they became dislodged from the bulk, and preventing the collagenase enzymes from entering the droplet and cleaving the biopolymers, which would have resulted in smaller fragments of polymer that may be able to diffuse across the alginate barrier.

Next, the barrier’s integrity was assessed after being injected through a 25 G hypodermic needle. As explained previously, one of the advantages of generating microtissues as opposed to a single, bulk macrotissue is the inherent injectability of the droplets. Therefore, it was essential that the protective alginate barrier formed around the particles maintained its integrity during injection. To evaluate the coating integrity, microtissues were injected and the thickness of the shell was measured (Figure 4b,c). As expected, individual microgels were able to dissociate from each other and move through a needle with minimal shear stresses. As a result, the injection process did not impact the microdroplets or their coating.

**Figure 5:**
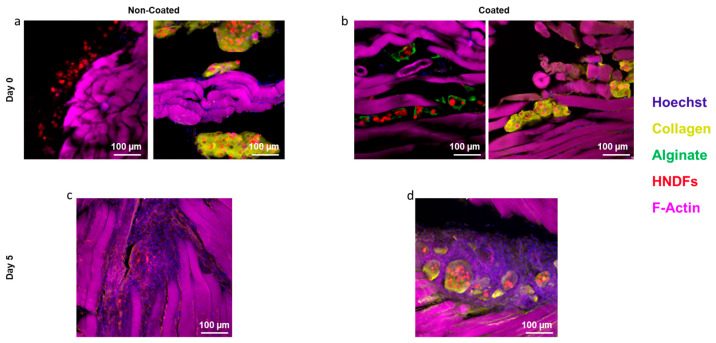
Injection of microtissues into in vivo murine model. (**a**) Immunofluorescent staining performed on Day 0 of the in vivo experiment confirms that the microtissues were successfully deployed within the murine muscle. (**b**) Immunofluorescent staining on Day 0 of the microtissues containing the protective alginate shell. (**c**) On Day 5, unprotected microtissues had largely been degraded and injected cells had escaped or been removed from the area. (**d**) Microtissues that contained a protective alginate coating maintained their integrity and, more importantly, maintained high cell concentrations at the site of injection. Scale bars = 100 µm.

Finally, as the motivation for this research was the formulation of protected microtissues to be used for regenerative medicine, microtissues were injected into the gastrocnemius of six-week-old, female C57/BL mice. The mice were sacrificed either immediately or five days post-injection, and the injection site was analyzed post mortem. In particular, immunofluorescent staining was performed to detect the presence of cell nuclei, F-actin (to stain native mouse tissue), collagen type I (the main component of the ECM hydrogel), and alginate. Additionally, the cells used in generating the microtissues were genetically modified to express red fluorescent protein (RFP) so that they could be readily viewed and distinguished from the native mouse cells. The success of the injection can be easily ascertained from the presence of cells and collagen on Day 0 (Figure 5a,b).

Five days after the surgery, the foreign body response (FBR) was easily observed by the recruitment of large numbers of cells to the site of the injection. While the FBR is a natural and beneficial response to injury, one of the challenges of working with microtissues is that macrophages and other cells summoned to the area during the FBR rapidly degrade the tissue and displace the injected cells [34]. Indeed, as expected, by Day 5, none of the initial ECM-based hydrogel was visible in the unprotected droplets, and the concentration of the injected, RFP-expressing cells is noticeably decreased (Figure 5c). On the other hand, the alginate-coated microtissues were largely unaffected by the FBR (Figure 5d). The ECM-based hydrogel was still present, and the RFP-expressing cells were clearly seen within the droplets. This result strongly suggests that the alginate barrier functions as hypothesized to protect cells and enhance their retention at the site of injection, which should make the use of these microtissues more beneficial to patients.

In particular, the protected microtissues described here are envisioned as a regenerative treatment option for patients needing implanted-cell therapies that do not require extensive tissue integration. That is, for conditions such as Parkinson’s disease (which may be treatable by implanting dopaminergic neurons) or diabetes (for which insulin-producing beta cells are required), the protection afforded by the alginate coating will provide a significant advantage over unprotected droplets.

## 3. Conclusions

We have developed a method for creating a protective barrier around microtissues based on the interaction between nanoparticles of calcium carbonate, encapsulated within the tissues, and alginate, which is externally added to them. Because the nanoparticles can be incorporated without reducing the volume available to cells and without changing the mechanical properties of the cells’ microenvironment, the entire process was shown to be fully biocompatible and even demonstrated a potential benefit to the cell viability. Furthermore, the two-step process we developed naturally lends itself to customized processing, as each step along the way can be independently modified to gain full control over both the microtissues and their protective barrier. Additionally, we have demonstrated in both in vitro and in vivo models that the barrier prevented both hydrolytic and enzymatic degradation of the microtissue’s ECM, which suggests that it could be utilized in tissue engineering applications that require an extended time for the cells to integrate into the host tissue. At the same time, we have demonstrated orthogonal control of the barrier’s degradation, and future work may include applications in which alginate lyase is encapsulated within the microtissue during the microfluidic fabrication in order to create a controlled degradation gradient for the barrier. Because of their inherent injectability and modularity, and because of how effectively they promote oxygen transport, microtissues promise to provide novel solutions to the challenges of regenerative medicine, and our technology, by providing more versatility and control over the long-term integrity of the tissues, moves this important area of research one step closer to clinical applicability.

## 4. Materials and Methods

ECM-based hydrogel preparation: Omental tissues were decellularized according to previously published protocols [8]. Briefly, porcine tissues (Kibbutz Lahav, Israel) were washed with phosphate-buffered saline (PBS), then incubated in a hypotonic buffer consisting of 10 mM Tris @ pH = 8 (Sigma-Aldrich; Rehovot, Israel), 5 mM ethylenediamine tetra-acetic acid (EDTA) (Sigma-Aldrich), and 1 μM phenylmethanesulfonyl-fluoride for 1 h. The tissue was then frozen and thrice thawed in the hypotonic buffer and gradually washed with 70% (*v*/*v*) ethanol followed by 100% ethanol for a half-hour each. Lipids were extracted via three half-hour washes with 100% acetone, followed by a 24 h soak in a 60:40 volumetric ratio of a solution of hexane and acetone (exchanged thrice during the 24 h). The tissue was next washed in 100% ethanol for a half-hour, transferred to 70% ethanol, and left to soak. The next day, the tissues were washed with PBS four times then soaked overnight in a solution of 0.25% trypsin–EDTA solution (Biological Industries; Beit Haemek, Israel). The tissue was thoroughly washed with PBS and soaked in a solution of 1.5 M NaCl (exchanged three times) overnight, at which time it was washed in a solution of 50 mM Tris and 1% Triton-X100 (Sigma-Aldrich) for 1 h. The decellularized tissue was finally washed in PBS, then DDW and then frozen (−20 °C) and freeze-dried.

Following this, the dry, decellularized omental tissue was ground into a powder with a Wiley Mini-Mill (Thomas Scientific; Swedesboro, NJ, USA). The omentum powder was digested enzymatically for 96 h at RT while stirring in a solution of 1 mg/mL pepsin (Sigma-Aldrich, 4000 U/mg) in 0.1 M HCl. Once enzymatic digestion was completed, the pH was adjusted to 7.4 by titration with 5 M NaOH, and concentrated Dulbecco’s modified Eagle medium (DMEM) was added until the concentration specified by the manufacturer was reached (Biological Industries). The concentration of decellularized omentum in the final solution was 1% (*w*/*v*).

Microfluidic device generation: Soft lithography techniques were used to fabricate the microfluidic devices. Negative photoresist SU-8 (3050, MicroChem Corp.; Newton, MA, USA) was first spin-coated onto a clean silicon wafer with a thickness of 300 μm (University Wafer; Boston, MA, USA) to either a thickness of 50 or 75 μm, and this was then patterned by UV exposure through a transparency photomask. Next a 10:1 ratio of PDMS prepolymer and curing agent (Sylgard 184, Dow Corning Corp.; Midland, MI, USA) was poured onto the master wafer. After one hour of curing at 65 °C, the replicas were extricated from the master wafer. Inlet holes and outlet holes were punctured via a 0.75 mm diameter biopsy punch (World Precision Instruments; Sarasota, FL, USA). Oxygen plasma (Diener Electronic GmbH & Co; KG; Ebhausen, Germany) was then used to permanently adhere the PDMS replicas to each other. Finally, the device channels were washed using Aquapel (PPG Industries; Pittsburgh, PA, USA) and immediately air-dried.

Droplet generation with CaCO_3_ nanoparticles: A suspension of 10 mg/mL of 50 nm calcium carbonate nanoparticles (^np^CaCO_3_) (US Research Nanomaterials Inc.; Houston, TX, USA) was mixed with 1% (*w*/*v*) omentum hydrogel in a 1:5 ratio, yielding a final omentum concentration of 0.83% (*w*/*v*) and a final concentration of ^np^CaCO_3_ of 0.16% (*w*/*v*). This mixture was then passed through a 70 µm nylon strainer (Corning; Airport City, Israel) and loaded into a plastic, 1 mL syringe. A mixture of 2% Pico-Surf 2 surfactant in Novec-7500 oil (Sphere Fluidics; Cambridge, UK) was also loaded into a plastic 1 mL syringe for use as the outer phase. Two fine-bore polythene tubes (Smiths Medical International Ltd.; Ashford, UK) with an outer diameter of 1.09 mm and an inner diameter of 0.38 mm were used to connect the syringes to the device inlets. Flow rates were controlled by an NE-1000 syringe pump (New Era Pump Systems; Farmingdale, NY, USA). Droplet generation was monitored using a digital microscope (Dino-lite Digital Microscope; New Taipei City, Taiwan). Flow rates of 100 µL/h (oil phase) and 50 µL/h (hydrogel phase) were used to obtain the desired droplet size of either 65–85 µm or 110–120 µm depending on the thickness of the device’s channels. The system was operated at room temperature. Generated droplets were collected in an Eppendorf tube for 15 min and transferred immediately to 37 °C for 15 min to allow gelation.

After gelation, droplets were mixed with 50 µL of 20% (*v*/*v*) 1H,1H,2H,2H-perfluoro-1-octanol (Sigma-Aldrich, SKU 370533) in perfluoro-compound FC-40 (Sigma-Aldrich), which serves as an emulsion destabilizer. Finally, 600 µL of Dulbecco’s modified Eagle medium (DMEM) was added to the tube to transfer the droplets to an aqueous phase.

Droplet generation with liposomes: A 1% (*w*/*v*) ECM-based omentum hydrogel was mixed in a ratio of 1:10 with FITC liposomes (provided by Dr. Dan Peer’s lab, Tel Aviv University). The mixture was passed through a 70 µm nylon strainer and loaded into a plastic 1 mL syringe. The same process described above was then carried out on the hydrogel–liposome mixture.

Cell Culture: NIH/3T3 fibroblast cells (ATCC) were grown in DMEM supplemented with a 10% (*v*/*v*) fetal bovine serum (FBS) (Biological Industries), 1% (*v*/*v*) L-glutamine (L-Glu) (Biological Industries), and 1% (*v*/*v*) penicillin/streptomycin (Biological Industries). Upon reaching approximately 80% confluence, cells were passaged for expansion under sterile conditions and incubated at 37 °C in a humidified, 5% CO_2_ incubator. Subculture was carried out by washing with PBS for 30 s and incubating in 0.05% trypsin/EDTA for 3 min.

Red fluorescent protein–human neonatal dermal fibroblasts (RFP-HNDFs) (Lonza; Haifa, Israel) were grown in DMEM supplemented with 10% (*v*/*v*) FBS, 1% (*v*/*v*) penicillin/streptomycin, 1% L-Glu, MEM non-essential amino acids solution (Gibco; Paisley Scotland), and 0.2% β-mercaptoethanol 50 mM (Gibco; Paisley, Scotland). At approximately 80% confluence, cells were passaged for expansion under sterile conditions and incubated at 37 °C in a humidified 5% CO_2_ incubator.

Generation of microtissues with encapsulated cells: The 40 mg/mL ^np^CaCO_3_ suspensions were mixed with 1% (*w*/*v*) ECM-based hydrogel in a 1:20 volumetric ratio, resulting in a final concentration of 0.95% (*w*/*v*) hydrogel and 0.19% (*w*/*v*) ^np^CaCO_3_. The mixture was then passed through a 70 µm nylon strainer. Next, cells were suspended in seven times their volume of the filtered hydrogel mixture, leading to final concentrations of: 0.83% (*w*/*v*) hydrogel, 0.16% (*w*/*v*) ^np^CaCO_3_, and 20 × 10^6^ cells/mL (in the case of NIH/3T3 cells) or 25 × 10^6^ cells/mL (RFP-HNDFs). This mixture was loaded into a 1 mL syringe, and droplets with ^np^CaCO_3_ and cells were created by following the procedure described above. Prior to mixing with the hydrogel, cell viability was assessed by Trypan Blue (Biological Industries) exclusion, and the cells were counted and centrifuged at 600 RPM for 5 min to concentrate them to the desired volume.

Creation of alginate capsule: Alginate powder (LFR 5/60, LF 10/60 FT, or LF 200 FTS) (FMC BioPolymer; Philadelphia, PA, USA) was dissolved in MES buffer (pH = 6) to reach the desired concentration. The solution was filtered through 0.22 µm filters (Millex Syringe-driven Filter Units, Merck; Rehovot, Israel) and stored at 4 °C until use. Microgels were generated as described and collected in a 15 mL Falcon tube. Following collection, the droplets were concentrated by centrifugation at 1000 RPM for 10 min, and the concentrated droplets were gently suspended and added dropwise to a 3 mL alginate solution in a 6-well tissue culture plate on ice. Immediately following the addition of the droplets to the alginate solution, the plate was gently shaken for 30 min and was then washed twice with DMEM and centrifuged at 1000 RPM for 10 min.

Synthesis of alginate with CY5 fluorescent marker: A solution of 1% LFR 5/60 was prepared in 0.1 M MES buffer and added to a round bottom flask. A 5 M excess of 10 mg/mL ethylene dicarbodiimide (EDC, ThermoFisher, Waltham, MA, USA) solution was added dropwise and allowed to react for 1 hr. The pH was then raised to 8–8.5 by titration with triethylamine, and a 5 M excess of 10 mg/mL N-hydrosuccinimide (NHS) solution was added alongside an equivalent excess of a primer amine-CY5 fluorescent molecule that was a kind gift from Prof. Roey Amir. This reaction was allowed to proceed for two hours. The reaction mixture was dialyzed (SnakeSkin™ dialysis tubing, 10 kDa MWCO, 16 m, ThermoFisher Scientific) for 4 days against a 0.1 M NaCl solution which was refreshed twice daily. After dialysis, the solution was frozen in liquid nitrogen and lyophilized.

Preparation of alginate with fluorescent microparticles: First, 20% bovine serum albumin (BSA) in DDW was mixed with 1 µm polystyrene dark blue particles (Sigma-Aldrich) in a 1:10 volumetric ratio and left at room temperature for 30 min. The particles were then mixed at a 1:10 volumetric ratio with the alginate solution, and this mixture was used for coating the ECM-based hydrogel. The thickness of the coating was measured via an upright confocal microscope (Nikon ECLIPSE NI-E; Melville, NY, USA). For each droplet that was examined, the central plane of the droplet was determined by finding the midpoint between the top and bottom of the droplet in the Z-axis. The coating thickness was recorded in at least five different locations along the central plane, and these values were averaged to generate the estimated coating thickness for each droplet. The average thickness for a given condition is the average of at least 17 droplets.

Rheology: Calcium carbonate nanoparticles were suspended at concentrations of 0.5, 1, 2, 5, 10, or 40 mg/mL, and the suspension was mixed with 1% (*w*/*v*) hydrogel in a volumetric ratio of 1:5. Then, 80 µL of the mixture was loaded into a PDMS mold (that had been previously punched with an 8 mm puncher) and incubated at 37 °C in a humidified, 5% CO_2_ incubator for 1 h to allow the hydrogel to set. Next, gels were removed from the mold, exposed to a 1% alginate solution for 30 min, and washed with 0.1 M HEPES. Rheological measurements were performed using a Discovery HR-3 Hybrid Rheometer (TA Instruments; New Castle DE, USA) with 8 mm diameter parallel-plate geometry. The samples were loaded at a temperature of 37 °C, and their viscoelastic properties were measured by performing a frequency sweep between 0.01 and 10 rad/s at a constant 1% strain. At least three gels were assessed and averaged for each condition.

Live/Dead Assay: Cell viability within coated or non-coated droplets was determined by incubating cells in fluorescein diacetate (Sigma-Aldrich, 7 µg/mL) and propidium iodide (Sigma-Aldrich, 5 µg/mL) for fifteen minutes in a humidified incubator with 5% CO_2_, at 37 °C. Three samples of droplets were observed with an inverted fluorescence microscope (Nikon Eclipse TI). The numbers of live and dead cells were determined by manual counting using the NIS Elements software (Version 4.13) (Nikon; Melville, NY, USA) from at least three different microscopic field images in each sample. For each time point, cells were counted in 40 droplets.

Proliferation Assay: Encapsulated cells in either coated or non-coated droplets were placed in a 24-well tissue culture plate that was pre-coated with polyHEMA (Sigma; Rehovot, Israel). For each time point, three measurements were recorded per group. PrestoBlue™ reagent (Thermo Fisher) was added to each well at a 1:9 ratio with cell medium and incubated for 5 h. The absorbance was measured at 570 nm (600 nm serving as the reference wavelength) using a Tecan Plate Reader InfiniteM200pro. All values were normalized to Day 1.

Cell Escape: Five Eppendorf tubes of encapsulated cells were collected and merged into a pool of cellular droplets in a 15 mL Falcon tube. Later, the suspension of droplets was split into three groups: (1) coated droplets, (2) coated droplets with the addition of 1 U/mL of alginate lyase (Sigma-Aldrich), and (3) non-coated droplets. Groups 1 and 2 underwent the aforementioned coating process. All droplets then underwent three rounds of pre-plating for 30 min to remove non-encapsulated cells. Next, 10 µL from each group was placed in 6 different wells of a 24-well tissue culture plate, and 1 mL of medium was added to each well. The migration distance of cells outside the droplets was quantified based on brightfield (BF) images taken on Days 2, 3, and 5 by inverted fluorescence microscopy. For each image, the average migration distance outside the droplet was quantified by using the NIS Elements software (Nikon). Results were averaged over 50 droplets per group.

Degradation assay: NIH/3T3 fibroblasts cells were encapsulated to form microtissues. All droplets were pooled in a 15 mL Falcon tube. Half of the suspension was then coated. Both groups were then separately concentrated to a volume of 100 µL, resuspended gently, and evenly split into 4 wells (25 µL per well) of a 24-well tissue culture plate that had been previously coated with polyHEMA, and cell medium was added to reach a final volume of 500 µL per well. Next, 1 µL of 500 U/mL collagenase type II powder (Worthington; Lakewood, NJ, USA) solution was added to each well, achieving a final concentration of 1 U/mL. Results were compared against a control group without collagenase. Degradation was assessed at Days 0, 1, 2, and 3 by measuring the diameter of the droplets using the NIS Elements software (Nikon). The results presented represent the average of 50 droplets per condition.

In vivo testing: All mice were treated according to the ethical regulations of Tel Aviv University. Permission was granted by the ethics committee, protocol number 04-19-028. Six-week-old C57/BL female mice, purchased from ENVIGO (Jerusalem, Israel), were randomly divided into groups. Two mice were allotted to each group other than Day 0 for which only one mouse was used. The animals were anaesthetized subcutaneously with a ketamine (100 mg/kg)–xylazine (10 mg/kg) cocktail in saline solution. The gastrocnemius muscle of the mice was shaved in the area of the injection and either coated or non-coated droplets were injected.

Sixteen Eppendorf tubes of microtissues with encapsulated RFP-HNDF were generated and collected. The collected microtissues were pooled in a 15 mL Falcon tube and subsequently split into a coated group and a non-coated group. The coated droplets were coated with 1% alginate LF 200 FTS at pH = 6 for 30 min. The droplets (for each condition) were concentrated by centrifugation (300× *g* for 5 min) and resuspended in 600 µL of medium. The 600 µL suspension was divided equally into six Eppendorf tubes. Then, each tube’s 100 µL suspension was loaded into a 1 mL syringe with a 25 G needle and 60 µL was injected intramuscularly into the gastrocnemius muscle of each mouse.

In vivo tissue fixation and staining: Mice were sacrificed after one hour, two days, or five days. The gastrocnemius muscles were extracted, fixed in 4% formaldehyde, and embedded in optimal cutting temperature (OCT) compound (Tissue-Tek^®^ OCT Compound Perrigo). Using a Cryotome™ FSE (Thermo Scientific), 60 μm thick sections were prepared and affixed to X-tra^®^ adhesive glass slides (Leica Biosystems; Wetzler, Germany). The fixed samples were washed twice in PBS to extract the OCT compound and permeabilized with 0.05% (*v*/*v*) Triton X-100 (Sigma) in a blocking solution of PBS containing 1% BSA and 10% FBS for 1 h. The samples were incubated for 90 min with primary antibody Mouse αAlginate (1:200, Sigma 5200132), washed three times, and incubated for another 90 min with FITC-Goat-αMouse (1:250, Abcam; Cambridge, UK, ab150113), Phalloidin-iFluor 647 (1:1000, Abcam, ab176759) for immunostaining of actin filaments, and Hoechst 33258 (1:20; Sigma-Aldrich) for nuclei detection. The samples were washed three times, mounted, and visualized by upright confocal microscopy.

Statistical analysis: Statistical analysis was performed using the GraphPad Prism software (Version 8.4.2). All values are given as mean ± SEM. For experiments with only two groups, a *t*-test was performed. We applied one-tail standards for experiments in which we hypothesized that the alginate-coated droplets would tend to a particular outcome. In experiments without an obvious outcome, two-tail standards were applied. For experiments with three groups or more, one- or two-way ANOVA was used as appropriate. For multiple comparison ANOVA analyses, the Tukey post hoc test was applied unless otherwise stated. Statistical significance was set at *p* < 0.05.

## Data Availability

The data presented in this study are available on request from the corresponding author.
